# Nutritional status of patients occupying ICUs in the state of Rio de Janeiro

**DOI:** 10.1186/cc10765

**Published:** 2012-03-20

**Authors:** SO Oliveira, R Goldwasses, U Melo, M Bandeira, F Dessa, R Almeida, I Kouh

**Affiliations:** 1 Albert Schweitzer State Hospital, Rio de Janeiro, Brazil; 2 Healh Provider State Government, Rio de Janeiro, Brazil; 3 Alberto Torres State Hospital, São Gonçalo, Brazil; 4 Real Cordis Hospital, Rio de Janeiro, Brazil; 5 Bangu Hospital, Rio de Janeiro, Brazil; 6 UFRJ University Hospital, Rio de Janeiro, Brazil

## Introduction

Nutritional status and anemia influence the clinical course of hospitalized patients. Anemia appears in the first days of hospitalization and can sustain itself and grow worse over time and is caused by a number of factors such as dilution secondary to fluid replacement, hemolysis, abnormalities in iron metabolism, blood loss in the gastrointestinal tract and also due to decreased production of erythropoietin, a consequence of decreased erythropoiesis caused by the presence of inflammatory cytokines.

## Methods

A cross-sectional study on 30 November, patients >18 years. Evaluated characteristics of all patients admitted with age, sex, APACHE II score, mean length of stay, cause of hospitalization in mechanical ventilation, organ failure, sedation and analgesia, coma and underuse of vasoactive drugs.

## Results

The study included 247 patients hospitalized in ICUs, mean age 63 years and 60% (148 patients) were male. Sepsis was the most frequent cause of hospitalization at 57% and the average hospital stay was 16 days. The rate of albumin and mean hemoglobin level were respectively 2.1 and 9.5 g/dl. For those patients hospitalized over 10 days were observed average levels of 1.5 and 8.9 g/dl. For mechanical ventilation in patients with septic shock the results were 1.4 and 7.9 g/dl with a mean hospital stay of 14 days. The postoperative group was the highest level observed at 2.6 and 10.4 g/dl and mean total time of hospitalization of 5 days. The worst results based on diagnosis were respectively pulmonary septic shock, ischemic hemispheric brain stroke and cardiogenic shock. All patients with length of stay over 11 days resulted in a clinically malnourished state.

## Conclusion

The age, length of stay and diagnosis associated with the level of organ dysfunction are key factors to progress to the state of malnutrition. The multidisciplinary team has an ongoing role in controlling the supply of proteins and calories with essential nutrients in order to improve the provision, preventing complications and adverse outcomes.
